# Supplementary material for: Moderate–Vigorous Physical Activity and Clinical Outcomes in Adults with Nondialysis Chronic Kidney Disease
Source: J Clin Med. 2021 Jul 29;10(15):3365. doi: 10.3390/jcm10153365 (PMC8347400; doi:10.3390/jcm10153365)
Supplement: Supplementary file 1 [file jcm-10-03365-s001.zip › jcm-1278988-supplementary.pdf]

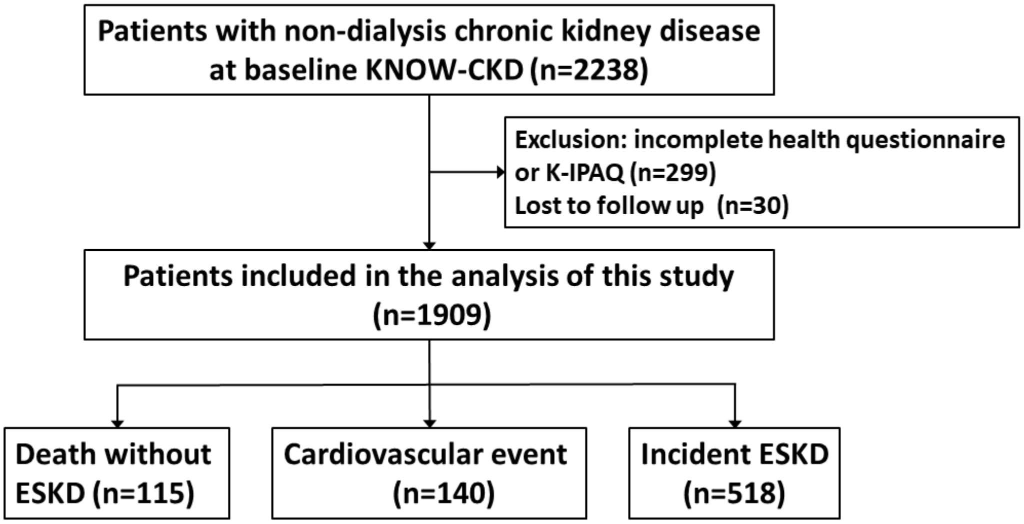

Figure S1. Flowchart demonstrates patient selection and clinical events. During a median observation period of 5.9 years, there were 518 incident ESKD, 140 CVE, and 115 all-cause deaths.

Data S1. The International Physical Activity Questionnaire Short Form (IPAQ-SF).

1. During the past 7 days, on how many days did you walk for at least 10 minutes at a time?
(This includes at work and school, walking to travel from place to place, and walking that you have done for exercise)
1-1. How much time did you usually walk on each of those days?

2. During the past 7 days, on how many days did you do moderate physical activities that take moderate physical effort or make you breathe somewhat harder than normal?
(Moderate physical activities: Job-related activities and sports like carrying light loads, slow swimming, double tennis, volleyball, badminton, or table tennis. Do not include walking)
2-1. How much time did you usually spend performing moderate PA on each of those days?

3. During the past 7 days, on how many days did you do vigorous physical activities that take hard physical effort and make you breathe much harder than normal? Think only about those physical activities that you did for at least 10 minutes at a time.
(Vigorous physical activities: Job-related activities and sports like carrying heavy loads, running, mountain climbing, fast bicycling, fast swimming, soccer, basketball, rope jumping, squash, or singles tennis)
3-1. How much time did you usually spend performing vigorous PA on each of those days?

4. During the last 7 days, on how many days did you do muscle-strengthening activities such as push-ups, sit-ups, lifting dumbbells or barbells, or working at an exercise bar?

5. During the past 7 days, on how many days did you do flexibility activities such as stretching, or doing calisthenics?
